# Viral interactions with host factors (TIM-1, TAM -receptors, Glut-1) are related to the disruption of glucose and ascorbate transport and homeostasis, causing the haemorrhagic manifestations of viral haemorrhagic fevers.

**DOI:** 10.12688/f1000research.134121.5

**Published:** 2024-10-28

**Authors:** Ivan Chicano Wust

**Affiliations:** 1 Universidad Nacional de Educacion a Distancia, Madrid, Community of Madrid, Spain

**Keywords:** Ebola virus, Lassa virus, Dengue virus, haemorrhagic fevers, glucose, ascorbate, oxidative stress

## Abstract

The haemorrhagic features of viral haemorrhagic fevers may be caused by common patterns of metabolic disturbances of the glucose and ascorbate homeostasis. Haemorrhages and vasculature disfunctions are a clinical feature not only of viral haemorrhagic fevers, but also in scurvy, diabetes and thrombotic microangiopathic haemolytic anaemia. Interestingly, the expression of glucose and ascorbate transporter Glut-1 on the erythrocyte membrane is associated with the inability to synthesize ascorbate and is restricted to that very species that are susceptible to filoviruses (primates, humans and fruit bats). Glut-1 may play a pivotal role in haemorrhagic fever pathogenesis. TIM-1 and TAM receptors have been recognized to enhance entry of Ebola, Lassa and Dengue viruses and viral interferences with TIM-1 could disturb its function, disturbing the expression of Glut-1. In those species not able to synthesize ascorbate and expressing Glut-1 on erythrocytes virus could interact with Glut-1 or other functionally related protein, and the influx of glucose into the cells would be severely impaired. As a consequence, transient hyperglycemia and a marked oxidative stress coupled with the high levels of glucose in plasma would be established, and then promote the activation of NF–κB transcription, exacerbating a pro-inflammatory response mediated by cytokines and chemokines: The inability to synthesize ascorbate is an Achilles Heel when trying to counteract the oxidative stress.

## The haemorrhagic features of viral haemorrhagic fevers are caused by common patterns of metabolic disturbances of the glucose and ascorbate homeostasis

In the present opinion article, it will be disclosed that the haemorrhagic features of viral haemorrhagic fevers in general, and those caused by Lassa virus, dengue virus and especially Ebola virus, in particular, are caused by common patterns of metabolic disturbances of the glucose and ascorbate homeostasis. Disruption of glucose and ascorbate homeostasis could be the reason of both enhanced inflammatory cytokine storming and haemorrhagic manifestations at the level of endothelial cells and vasculature injury. ^1^^–^^5^ Endothelial damage during filovirus infection with no evidence of direct endothelial cytolysis has previously been described, ^6^ reinforcing the idea that other indirect mechanisms governing vasculature injury are present. Intracellular ascorbic acid has been considered as an enhancer of nitric oxide (NO) synthesis ^7^ and the macromolecular permeability of cultured human umbilical vein endothelial cell monolayers was decreased significantly in culture medium containing L-ascorbic acid and L-ascorbic acid 2-phosphate. ^8^ Apoptosis in endothelial cells can be induced by hyperglycemia and ascorbate helps to prevent endothelial dysfunction, stimulates type-IV collagen synthesis and enhances cell proliferation. Haemorrhages and vasculature disfunctions are a clinical feature not only of viral haemorrhagic fevers, but also in scurvy, diabetes and thrombotic microangiopathic haemolytic anaemia. Inflammatory cytokine concentrations were found to be acutely increased by hyperglycaemia in humans, ^4^^,^^5^ which has been suggested as the causative role in the vasculature disorders and immune activation of diabetes. Hypoascorbinaemia and diabetes mellitus share several clinical symptoms including microangiopathy, capillary hyperperfusion and haemorrhages.

## Microangiopathic haemolytic anemia: a viral haemorrhagic fever without virus?

An early description by Symmers in 1952 of the thrombotic microangiopathic haemolytic anaemia, ^9^ which extraordinarily fits the haemorrhagic and vascular manifestations of filovirus haemorrhagic fever (FHF), included haemorrhagic manifestations in the form of widespread purpura. As described by Symmers, “ *there might be haemorrhage from any of the orifices of the body*”. A prodromal period of fever, muscle and joint pains and vague abdominal pain existed and included also mental confusion and neurological symptoms. The parallel of the thrombotic microangiopathic haemolytic anaemia with the filovirus pathological manifestations is important and would become more evident in its physiological implications basing on the hypothesis description of virus-driven biochemical events.

### Glucose and ascorbate metabolism as an Achilles’ heel in filovirus haemorrhagic fevers (FHF)

Several receptor candidates have been proposed as mediators for filovirus entry and several attempts to develop a vaccine have been pursued but there is no suitable definitive biological explanation of the violence of the illness manifestations, nor are well defined the factors underlying the differences in illness severity between viruses and within the species range. Whereas many experimental data are available and have rendered important descriptive and molecular information, there is not an integrative and comprehensive perspective. A better comprehension of the virus pathophysiology could help in developing a treatment strategy. According to literature major symptoms of Ebola virus disease include a maculopapular rash and mucosal haemorrhage. It begins unspecific with fever, asthenia, diarrhea, headaches, myalgia, arthralgia, vomiting and abdominal pain and disease course develops sharply and resolves in survival or death in the course of approximately 3 weeks. Massive bleeding along with fluid distribution problems, disseminated intravascular coagulation and focal necrosis are observed in fatal cases. Arthralgia, asthenia, and neurological disorders, as dysesthesias, are often sequelae in the convalescence, which lasts weeks to months. Main features of Ebola virus haemorrhagic fevers can be consulted in available literature. ^10^^–^^14^

Glucose transporter 1 or Glut 1, also known as solute carrier family 2, facilitated glucose transporter member 1 (SLC2A1), is a uniporter protein that in humans is encoded by the *SLC2A1* gene. ^15^ Glut 1 facilitates the transport of glucose across the plasma membranes of mammalian cells. ^16^ Glut 1 is responsible for the low level of basal glucose uptake required to sustain respiration in all cells. Glut 1 is also a major receptor for uptake of vitamin C (ascorbate), especially in non-ascorbate producing mammals as part of an adaptation to compensate by participating in a vitamin C recycling process. In mammals that do produce vitamin C, Glut 4 is often expressed instead of Glut 1. ^17^ Interestingly, expression of Glut-1 on the erythrocyte membrane is associated with the inability to synthesize ascorbate ^18^ and is restricted to that very species that are susceptible to filoviruses or are considered to be the reservoir of the virus in nature (primates, humans and fruit bats). Glucose and ascorbate metabolism represent probably an Achilles’ heel in filovirus haemorrhagic fevers (FHF) and Glut-1 may play a pivotal role in haemorrhagic fever pathogenesis. In other words, Glut-1 on erythrocytes and inability to synthesize ascorbate may account for the pathophysiology of filovirus haemorrhagic fevers in susceptible species. Ebola and Marburg viruses cause haemorrhagic fever in human and non-human primates. Guinea pigs are frequently used as an animal model after a trivial virus strain adaptation. Fruit bats are considered the reservoir of filoviruses in nature. ^19^ Extraordinary mechanisms regarding glucose absorption and transport are displayed by some fruit bats in which serological evidence to Ebola virus was confirmed or Marburg virus was isolated. Pigs, ^20^^,^^21^ which develop symptomless infection by filoviruses differ from the inability of other species to synthesize ascorbate and the presence of Glut-1 on the erythrocyte membranes. Thus, filoviruses in those species expressing the glucose and ascorbate transporter Glut-1 on erythrocytes and unable to synthesize ascorbate, would cause pathophysiological changes that account for the severity of the filoviral haemorrhagic fevers, by means of a virus-driven disruption or overwhelming of ascorbate and/or glucose homeostasis. The inability to synthesize ascorbate could represent an Achilles’ heel in counteracting the exacerbated inflammatory response ^22^ during Ebola haemorrhagic fever. Transport of dehydroascorbic acid (DHA) from plasma is coupled with its reduction with glutathione (GSH) and NADH. Erythrocytes, with the highest level of Glut-1 expression, regulate the concentrations of ascorbate in plasma by the influx of DHA and efflux of DHA and ascorbate. Glucose and DHA compete for the same transporter and as plasma concentrations of D-glucose are quite higher than DHA concentrations, DHA uptake is more important when ascorbate is rapidly oxidized outside the cells, much like in areas of inflammation, as during FHF.

### *In vitro* evidence about interactions of Glut-1 with filovirus glycoproteins

**Figure 1.**
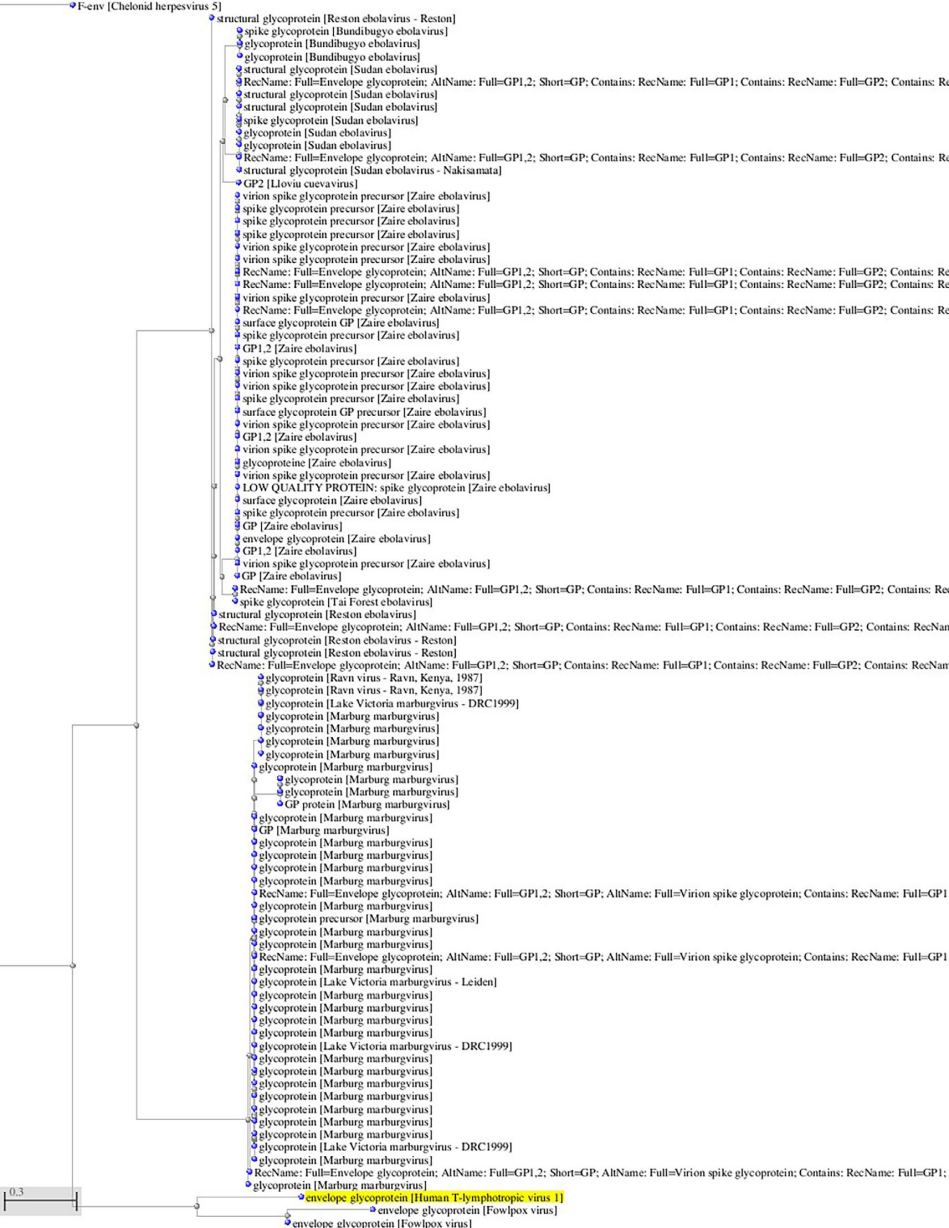
A, B. Basic Local Alignment Search Tool blastp 2.2.28+ for non-redundant protein sequences (restricted to viruses and excluding retroviruses) with significant homology to the glycoprotein sequence of HTLV-1 (GenBank AAB42125 173aa). Tree views by the neighbor joining method of the aligned sequences (Blastp 2.2.28+). Fast minimum evolution. Max sequence difference, 0,85, Grishin distance.

Viral interactions of filoviruses, or interferences, with Glut-1, specially on the erythrocyte membrane could be hypothesized. This might account for the pathophysiological changes during infection. In favor of direct glycoprotein interactions with Glut-1 are the experiments demonstrating inhibition of Ebola-glycoprotein mediated entry by cytokalasin B, ^23^ which is a potent inhibitor of glucose and DHA influx transport in mammalian cells and rather than as agent impairing microfilament function should be considered as an inhibitor of Glut-1, having being used as such for studies on HTLV-1 and HTLV-2 entry, ^24^ viruses that use Glut-1 as a receptor for virus entry. Interestingly, there is some sequence homology between HTLV-1 and HTLV-2 glycoproteins and filoviruses glycoprotein (see Figure 1). As cytokalasin B exerts its inhibitory effect by docking at the positively charged endofacial pocket 3 A of Glut-1, ^25^ mutagenesis of this site should be tested and its influence on Ebola virus glycoprotein interactions and entry into the cell. Also in favor of direct glycoprotein interactions with Glut-1 are the facts that Phosphoinositide-3 kinase-Akt pathway controlls celular entry of Ebola virus ^26^: inhibition of PI3K, Akt or Rac1 disrupted normal uptake of virus particles into cells; and cytokine stimulation promotes glucose uptake via phosphatidil inositol-3 kinase/Akt regulation of Glut-1 activity and trafficking ^27^: Inihibition of mTOR/RAPTOR by ripamycin greatly diminished glucosa uptake suggesting Akt-stimulated mTOR/RAPTOR may promote Glut-1 transporter activity.

On the other hand, the integral protein stomatin, in the lipid rafts of the erythrocyte, regulates the switch from glucose to DHA transport, regulating the substrate preference of the transporter. ^16^ Stomatocytosis, after which the protein stomatin is named, is a haemolytic condition with substantial intravascular haemolysis.

### TIM-1 receptor and TAM family of receptors as a link between viral interactions with host proteins, haemorrhagic manifestations of viral haemorrhagic fevers and disruption of glucose and ascorbate transport and homeostasis

Ebola virus has very broad cell tropism and it may bind to multiple attachment factors, among which are numerous lectins (DC-SIGN/L-SIGN, MGL, LSECtin and β1 integrins. Along with these attachment factors three main receptor candidates have been proposed as mediators for Ebola virus entry:

- NPC1 was identified as an endosome-associated receptor for filovirus entry. This protein is required for filovirus entry and confers susceptibility to filovirus infection when expressed in non-permissive reptilian cells. ^28^
- The TAM receptor Axl, which has been proven to enhance Ebola virus particles macropinocytosis. ^29^
- TIM-1. ^30^^,^^31^ It was published that T-cell Ig and mucin domain 1 (TIM-1) binds to the receptor binding domain of the Zaire Ebola virus (EBOV) glycoprotein, and ectopic TIM-1 expression in poorly permissive cells enhances EBOV infection by 10- to 30-fold.

Interestingly, it has recently been published that TIM-1 mediates also a dystroglycan independent entry of Lassa virus. ^32^ It has been pointed out that “a growing number of enveloped viruses are now appreciated to enter some cell populations through phosphatidylserine (PtdSer) receptor interactions”. ^33^^,^^34^ The TIM receptors directly interact with PtdSer on apoptotic bodies or membrane-associated viruses, ^35^^–^^38^ whereas the TAM receptors bind to one of two serum proteins, Gas6 or protein S, which, in turn, bind to PtdSer. ^39^^–^^41^

Regarding other virus associated with haemorrhagic manifestations, as is dengue virus (DENV), the same TIM and TAM families of phosphatidylserine receptors have been found to mediate dengue virus entry. ^42^ Thus, TIM-1 has been recognized to enhance entry of Ebola virus, dengue virus and Lassa virus, that are characterized by the haemorrhagic manifestations in the illness they cause.

And it has recently been found that TIM-3 expression decreases Glut 1 expression in Jurkat T cells at resting state and at an early time point of activation. ^43^ TIM-1 is preferentially expressed on Th2 cells and has been identified as a stimulatory molecule for T-cell activation. ^44^ TIM-3 is preferentially expressed on Th1 and Tc1 cells and functions as an inhibitory molecule, which mediates apoptosis of Th1 and Tc1 cells. ^45^ TIM-4 is preferentially expressed on antigen-presenting cells, modulating the phagocytosis of apoptotic cells by interacting with phosphatidylserine (PS) exposed on apoptotic cell surface. ^35^ In addition to TIM-1, TIM-4 has been shown to augment Ebola virus entry comparably to TIM-1. ^46^

A key point of the present opinion is that TIM-1, a host factor that has been found to be receptor for Lassa virus, dengue virus and Ebola virus, could increase the expression of Glut-1 and that there could be a link between glucose and ascorbate transport, viral interactions with TIM-1 and haemorrhagic manifestations of haemorrhagic fevers. Currently it has been described influence of TIM-3 on the expression of Glut 1 but not for TIM-1. On the other hand the mechanism responsible of the haemorrhagic manifestations of haemorrhagic fevers is an unclear matter and no general features have yet been described of a general pathophysiological mechanism. In relation with their natural function, as TIM-1 is a stimulatory molecule for cell activation and TIM-3 functions as an inhibitory molecule, and considering that TIM-3 has been found to decrease Glut 1 expression in Jurkat T cells, it is to expect that TIM-1, in the inverse direction as it does TIM-3, increases the expression of Glut-1. This should be matter of further experimental research. As the key factor TIM-1 would enhance the expression of Glut-1 and the glucose and ascorbate transport, viral interferences with TIM-1 could disturb its function and disturb the expression of Glut-1 or disturb the ascorbate and glucose transport. This would imply a connection between Glut-1, glucose and ascorbate transport, haemorrhagic manifestations, which are linked to oxidative stress, disruption of glucose and ascorbate homeostasis, and the role of TIM family members as mediators of viral entry in those viruses that cause haemorrhagic syndromes and haemorrhagic fevers. If direct interactions of filoviral glycoproteins with Glut-1 could be demonstrated, it could partially explain the differences in severity of the disease manifestations in Ebola and Lassa infections and others.

On the other hand, TAM receptors, as is Axl, have been found to enhance entry of Ebola and dengue virus and it has been recently confirmed that endogenous expression of Axl does not actually enhance viral entry of Lassa virus (LV) in the presence of fully functional alpha dystroglycan receptor (α-DG receptor) but it strongly augments viral infection in the absence of α-DG. ^47^ And it has been described that Gas6–Axl receptor signalling is regulated by glucose in vascular smooth muscle cells. ^48^ The direct or indirect interaction of Axl with Glut-1 could be hypothesized basing on this finding that the physiological function of Axl is regulated by glucose. Akt-mTOr promotes Glut-1 transporter activity and recycling and can prevent Glut-1 internalization ^27^ and, according to Cavet and colleagues, ^48^ glucose exerted powerful effects on Gas6–Axl signaling, with greater activation of Akt and mTOR in low glucose and greater activation of ERK 1 /2 in high glucose. Akt-mTOr has been proven as key regulator of Zaire Ebola virus entry and activation of Ras-MAPK pathway, of which ERK1/2 is an activator, stimulates Ebola virus production from persistent infection. ^49^ These findings add to the hypothesis of the pivotal role of glucose, transporters and plasma levels in Filovirus disease.

## Concluding remarks and future perspectives

Glucose and ascorbate homeostasis, the presence of Glut-1 on erythrocytes and the erythrocyte physiology ^50^ might play an important role in Filovirus disease, not excluding viral interactions with the erythrocyte membrane. The blood glucose concentration is maintained within narrow limits by an inter-play between tissue glucose uptake, hepatic glucose production and insulin production. Erythrocytes, because of their number in blood, perform an important buffering function of glucose and ascorbate/dehydroascorbate levels in plasma. Erythrocyte counts of frugivorous species have been shown lower than those reported for insectivorous bats. ^51^ In those species not able to synthesize ascorbate and expressing Glut-1 on erythrocytes virus interactions would lead to severe disturbance of the glucose and ascorbate levels in plasma, activation of hypoxia-inducible factors and haemolysis. Coagulation abnormalities and haemorrhages are a clinical feature of Ebola virus haemorrhagic fever. It should also be tested the possible importance of haemolysis and erythrocyte-virus interactions. As it is shown in an important paper ^52^ erythrocytes can regulate platelet reactivity directly throughchemical signalling and adhesive erythrocyte-platelet interactions. Cell free hemoglobin induces platelet aggregation contributing to high risk of thrombotic complications. Adhesion of abnormal and/or stimulated erythrocytes to vascular endothelium can contribute to vasculature occlusions associated with thrombosis. Efficient blood coagulation requires sufficient pro-thrombotic surfaces, which are provided by cells that expose phosphatidylserine, which is normaly in the cytoplasmic side of the membrane to separate this pro-coagulant surface from plasma coagulation factors. The questions are: Could virus-erythrocytes interactions induce the exposure of phosphatidylserine on the cell surface? Is the phosphatidilserine-enriched envelope of some haemorrhagic fever-causing virions involved in this pro-thrombotic process mediated by exposed phosphatidylserine?

As the filovirus glycoproteins could interact with Glut-1 or other functionally related protein, the influx of glucose into the cells is severely impaired. As a consequence, transient hyperglycemia and a marked oxidative stress coupled with the high levels of glucose in plasma would be established, and then promote the activation of NF–κB transcription, exacerbating a pro-inflammatory response mediated by cytokines and chemokines, as described by Katherine Esposito et al. ^4^ On the other hand, the inability to synthesize ascorbate and the unavailability of ascorbate to entry into the cells through Glut-1 is an Achilles Heel when trying to counteract the oxidative stress. Additionally, there is another pathophysiological process to be mentioned: interactions with Glut-1 or other functionally related protein lead to an imbalance in the glucose homeostasis and the availability of glucose, not only for the erythrocytes, but also for CD8+ and CD4+ cells. These phenomena result in the bystander apoptosis of lymphocytes mediated by the lack of glucose, as described by Maclver et al. ^53^ Activated T cells have dramatically increased metabolic requirements to support their demands. ^54^ Therefore, activation of T cells causes a large increase in Glut-1 expression and surface localization, also promoting the Rab11b recycling of Glut-1 intracellular pools. ^53^ A work on the mechanisms and consequences of Ebolavirus-induced lymphocyte apoptosis concluded undefined that Ebola virus induces multiple pro-apoptotic stimuli. ^55^ As identified by Zhao and colleagues ^56^ in 2007 there is a glucose-initiated signaling pathway that leads to inhibition of GSK-3 and prevents cell death through stabilization of the anti-apoptotic BcI2 family protein McI. Normally targeted for proteasomal degradation by the ubiquitin E3 ligases, in highly glycolytic cells as are cancer cells and activated T cells, McI remains unphosphorylated and is not degraded, increasing the threshold for cell-death and maintaining cell survival. Conversely, when glucose uptake is limited, glycolytic flux decreases to a level that no longer sustains viability and pro-apoptotic signals promote cell death. ^53^

At a strictly cellular level, cell–adhesion dependent membrane trafficking of a binding partner for the Ebola virus glycoprotein has been shown to be a determinant of viral entry ^57^ and attachment to adhesion substratum has been shown to induce the accumulation of glucose transporters and stimulate glucose metabolism in PC12 cells. ^58^ According to Dube and colleagues a membrane trafficking event translocates the unknown binding partner of the receptor binding region (RBR) of Ebola glycoprotein (GP) to the cell surface and they identified two adherent primate lymphocytic cell lines that bind viral glycoprotein RBR at their surface and supported GP-mediated entry and infection. Lymphocytes are normally described in literature as non-permissive for Ebola-GP mediated entry and infection, ^59^ and activation of lymphocytes could be the trigger that renders them susceptible. According to the present hypothesis, this binding partner related to lymphocyte activation may possibly be or be related to Glut-1.

This first clue about a link between Ebola virus species range, expression of Glut-1 on erythrocytes and inability to synthesize ascorbate in humans and non-human primates should be further analyzed. In contrast to primates, and according to a study by Cui et al., bats are perhaps in the process of large-scale loss of ascorbate biosynthesis ability, ^60^ and show varying degrees of lack of gluconolactone oxidase function. This evolutionary recent loss of the ability to synthesize ascorbate could account for differences in Glut-1 expression and ascorbate metabolism resulting in differences in filovirus pathophysiology between species. In another study the same authors showed that the fruit bat *Rousettus leschenaultii* has retained the ability to synthesize ascorbate although at low levels compared with the mouse. ^61^ Olival et al. found serological evidence to both species Reston and Zaire of the genus Ebolavirus in *Rousettus leschenaultii*, one of the fruit bats species considered to be reservoir of Ebola virus, ^62^ and Marburgvirus has even been isolated from *Rousettus aegyptiacus*, ^63^ another Pteropodidae member. It is known that frugivorous and nectarivorous bats are able to ingest large quantities of sugar in a short time span while avoiding the potentially adverse side-effects of elevated blood glucose, which could be an important fact supporting the hypothesis that glucose and ascorbate metabolism account for the severity of the haemorrhagic fevers in primates and for the role of fruit bats as reservoir species maintaining low levels of filovirus replication. This ability to ingest large quantities of sugar in a short time span while avoiding the potentially adverse side-effects of elevated blood glucose has been related to the adaptive evolution in the glucose transporter 4 (Glut-4) gene in skeletal muscle ^64^ and also to high passive paracellular absorption of glucose in the gut of *Rousettus aegyptiacus*. ^65^ The importance of Glut-4 and the active sodium-ascorbate co-transporters (SVCTs) and active sodium-dependent glucose cotransporters (SGLTs) in regard to Filovirus pathophysiology should be further investigated.

Regarding Lassa fever virus, the differences in glucose and ascorbate physiology could be an explanation of the absence of haemorrhagic manifestations of the illness in mice while primates and humans develop haemorrhagic fevers. If direct interactions of filoviral glycoproteins with Glut-1 could be demonstrated, it could partialy explain the differences in severity of the disease manifestations between Ebola and Lassa infections and others. It has been shown that the Ebola virus glycoprotein is the main determinant of cell cytotoxicity and injury. ^66^ It should be further researched which role plays the viral glycoprotein in cell cytotoxicity for Lassa and Dengue viruses. The differences in haemorrhagic fever prevalence and severity between Ebola, Lassa and Dengue viruses could be explained by the differences in the pools of cellular factors and receptors involved in virus activity. Direct interactions with Glut-1 could explain the direct incidence of the Ebola virus on the glucose and ascorbate transport and homeostasis, causing the transient hyperglycemia, cytokine storming and haemorrhagic features. On the other hand, TIM-1 and Axl are entry factors for Lassa virus only under specific circumstances: in other words, are only a dystroglycan-independent entry route. The concentration of phosphatidylserine displayed in the viral envelope influences virus binding via Axl, as is described in another important paper. ^67^ According to these authors, since functional α-dystroglycan is recognized by the viral envelope protein GP1 of Lassa virus, the exact ratio of Lassa GP1 to phosphatidylserine in the viral envelope appears critical for receptor use. When the presence of phosphatidylserine on the virion surface is qualitatively important in relation to the expression of GP1 in Lassa virus, the cellular entry of the virus through the interaction phosphatidylserine-Gas6-Axl or phosphatidylserine-TIM-1 would lead to the hypothesized metabolic disturbances, mediated by TIM-1 and/or Axl, probably via Glut-1. When Axl or TIM-1 are not an important receptor for Lassa virus entry and entry occurs via interaction of the viral GP1 with α-dystroglycan, the incidence of metabolic disorders and haemorrhagic manifestations would be lower.

For Dengue virus, when virus-host interactions mainly take place via viral glycoprotein-DC-SIGN interaction, the metabolic glucose and ascorbate disorders and haemorrhagic manifestations would be less important than in those cases in which viral interactions with host factors involve the interaction of the viral glycoprotein with Axl and/or TIM-1. These different host factors that can interact with the viral glycoproteins would explain the differences in pathophysiology: normal Dengue fever when DC-SIGN-viral glycoprotein interactions are predominant, or Dengue severe haemorrhagic fever when Axl and/or TIM-1 interact predominantly with the dengue virus glycoprotein. The presence and importance of phosphatidylserine in the virus envelope could vary from virus to virus and from one virus type or strain (DENV-1, DENV-2, DENV-3, DENV-4, LV-1, LV-2, LFV-3, L V-4) to another. This should be matter of further research. It would be interesting to know which factors are governing the significant presence of phosphatidylserine on the virion envelope, and this considering the four haemorrhagic viruses: Ebola virus, Marburg virus, Lassa virus and Dengue virus.

Pathogenesis of viral haemorrhagic fevers is not yet well understood. The interaction of viruses with metabolism is a matter that will gain more attention in the next years. If some assumptions hypothesized in this opinion article were correct, regardless of other means of challenging the viruses, supportive care of haemorrhagic fevers almost in general could be significantly improved. It encompasses the use of insulin, ascorbate, glutathione (which participates, along with NADH, in the reduction of dehydroascorbate to ascorbate), maintaining constant and normal levels of glucose in plasma and general measures to counteract oxidative stress.

## Data Availability

No data are associated with this article.
