# Supplementary material for: Viral interactions with host factors (TIM-1, TAM -receptors, Glut-1) are related to the disruption of glucose and ascorbate transport and homeostasis, causing the haemorrhagic manifestations of viral haemorrhagic fevers
Source: F1000Res. 2024 Dec 9;12:518. Originally published 2023 May 18. [Version 6] doi: 10.12688/f1000research.134121.6 (PMC11809632; doi:10.12688/f1000research.134121.6)
Supplement: Peer review history

# Reviewer report: Reviewer response for version 6

**DOI:** 10.5256/f1000research.175501.r346870

**Published:** 2024-12-26

**Authors:** Hong-Li Liu

**Affiliations:** 1 Xi'an People's Hospital, Xi'an, China

I have read the revised manuscript and better understand the paper. The authors made an appropriate and sufficient revision, and I will approve the indexing of this paper. Thanks a lot and have a good day.

Is the topic of the opinion article discussed accurately in the context of the current literature?

Partly

Are arguments sufficiently supported by evidence from the published literature?

Yes

Are all factual statements correct and adequately supported by citations?

Yes

Are the conclusions drawn balanced and justified on the basis of the presented arguments?

Partly

Reviewer Expertise:

NA

I confirm that I have read this submission and believe that I have an appropriate level of expertise to confirm that it is of an acceptable scientific standard.

# Reviewer report: Reviewer response for version 5

**DOI:** 10.5256/f1000research.173777.r339488

**Published:** 2024-11-29

**Authors:** Chao Qin

**Affiliations:** 1 University of Southern California, Los Angeles, California, USA

This manuscript described a the correlation of metabolic receptors and virus induced diseases. It is a good topic to discuss. I would like the authors to address following issues in the manuscript.

 1. Please use more condensed languages for the statements, as well as the titles and subtitles.

 2. Please spell out the acronym at the first time of using it. 

 3. The original function of metabolic receptors such as GLUT1 should be explained in more details, as well as the role of glycolysis in filovirus infection. This will highlight the unique role GLUT1 in viral infection.

Is the topic of the opinion article discussed accurately in the context of the current literature?

Yes

Are arguments sufficiently supported by evidence from the published literature?

Yes

Are all factual statements correct and adequately supported by citations?

Yes

Are the conclusions drawn balanced and justified on the basis of the presented arguments?

Yes

Reviewer Expertise:

Metabolism; Viral infection; Immunology

I confirm that I have read this submission and believe that I have an appropriate level of expertise to confirm that it is of an acceptable scientific standard.

# Response

**Published:** 2024-12-19

**Authors:** Ivan Chicano Wust

**Affiliations:** -, Universidad Nacional de Educacion a Distancia, Madrid, Community of Madrid, Spain

Thank you for appreciating my effort and giving me some clues to improve the article.

# Reviewer report: Reviewer response for version 5

**DOI:** 10.5256/f1000research.173777.r339493

**Published:** 2024-11-29

**Authors:** Hong-Li Liu

**Affiliations:** 1 Xi'an People's Hospital, Xi'an, China

1. Rational use of punctuation to simplify sentence structure which makes it easy for readers to get the point of the paper;

 2. What is the most important purpose of this paper? The viral hemorrhagic fever or filovirus-related hemorrhagic fever? If the purpose is the latter, what is filovirus？which viruses are included in filovirus? it will be better for the author to make an introduction;

 3. The title talks about the relationship between the TIM-1, TAM, Glut-1, and the manifestation of viral hemorrhagic fever, and the Lassa virus, and dengue virus were mentioned in the text. If the authors want to present the relationship between these receptors and viral-related hemorrhagic fever. Actually, the Hantavirus is also a major agent of hemorrhagic fever that needs to pay attention;

 4. As the authors pointed out "the manifestations of thrombotic microangiopathic hemolytic anemia were similar to those of filovirus hemorrhagic fever", why did the authors mention this content? Or the TIM, Glut-1 is associated with this disorder?

 5. The concluding remarks should be precise and clear. The conclusion part is a summary but not a stacking of references or it will be hard for readers to get the main point of the paper;

 6. It is necessary for authors to reconstruct the manuscript. From the main topic, the introduction of receptor-related biological functions, the viruses that can cause hemorrhagic fever, and the relationship between these two parts involving the  possible pathways or mechanisms, and then conclude that  the pivotal role of these receptors in the viral-related hemorrhagic fever and their possible value in the treatment of these diseases.

Is the topic of the opinion article discussed accurately in the context of the current literature?

Partly

Are arguments sufficiently supported by evidence from the published literature?

Yes

Are all factual statements correct and adequately supported by citations?

Yes

Are the conclusions drawn balanced and justified on the basis of the presented arguments?

Partly

Reviewer Expertise:

infectious disease; HFRS; immunology

I confirm that I have read this submission and believe that I have an appropriate level of expertise to confirm that it is of an acceptable scientific standard, however I have significant reservations, as outlined above.

# Response

**Published:** 2024-12-19

**Authors:** Ivan Chicano Wust

**Affiliations:** -, Universidad Nacional de Educacion a Distancia, Madrid, Community of Madrid, Spain

Thank you for giving me the opportunity to improve the last version of the article.
